# Comparative analysis of commercial and “In-House” molecular tests for the detection of intestinal protozoa in stool samples

**DOI:** 10.1186/s13071-025-06879-9

**Published:** 2025-08-01

**Authors:** Giuseppe Di Pietra, Raffaele Gargiulo, Margherita Ortalli, Luciana Petrullo, Ester Oliva, Annibale Raglio, Annachiara Frigo, Ignazio Castagliuolo, Valeria Besutti

**Affiliations:** 1 Laboratory Medicine , ULSS 7 Pedemontana, Bassano del Grappa, Italy; 2 Department of Molecular Medicine, University of Padua, Padua, Italy; 3 Clinical Microbiology and Virology Unit, AOU Policlinico, Modena, Italy; 4 Unit of Microbiology, IRCCS Azienda Ospedaliero-Universitaria di Bologna, Bologna, Italy; 5 Unit of Microbiology, Cotugno Hospital–AORN “Ospedali dei Colli”, Naples, Italy; 6 Clinical Microbiology and Virology Unit, ASST Papa Giovanni XXIII, Bergamo, Italy; 7 Committee for the Study of Parasitology of the Italian Association of Clinical Microbiologists (CoSP-AMCLI), Milan, Italy; 8 Department of Cardio-Thoraco-Vascular Sciences and Public Health, Padua, Italy; 9 Microbiology and Virology Diagnostic Unit, Padua University Hospital, Padua, Italy

**Keywords:** *Giardia duodenalis*, *Cryptosporidium*, *Dientamoeba fragilis*, *Entamoeba histolytica/dispar*, Molecular diagnostics, Intestinal protozoa

## Abstract

**Background:**

Pathogenic intestinal protozoa exhibit a global distribution and are significant causes of diarrhea, estimated to affect approximately 3.5 billion individuals annually. These intestinal infections continue to pose formidable diagnostic challenges. Microscopy remains the reference diagnostic method for intestinal protozoa, but is limited in terms of sensitivity, specificity and the ability to differentiate closely related species. Additionally, microscopy requires an experienced microbiologist. Emerging diagnostic methods, such as immunochromatography and enzyme-linked immunosorbent assay (ELISA), are regarded as suitable techniques for rapid screening. Molecular diagnostic technologies, particularly real-time PCR (RT-PCR), are gaining traction in non-endemic areas characterised by low parasitic prevalence owing to their enhanced sensitivity and specificity, although these techniques still face various technical challenges.

**Methods:**

In this multicentre study involving 18 Italian laboratories, we compared the performance of a commercial RT-PCR test (AusDiagnostics) and an in-house RT-PCR assay against traditional microscopy for identifying infections with *Giardia duodenalis, Cryptosporidium* spp.,* Entamoeba histolytica* and *Dientamoeba fragilis*.

**Results:**

The study analysed 355 stool samples, of which 230 samples were freshly collected and 125 had been stored in preservation media. The data from our analyses show complete agreement between the AusDiagnostics and in-house PCR methods for the detection of *G. duodenalis*, with both methods demonstrating high sensitivity and specificity, similar to those of conventional microscopy. For *Cryptosporidium spp.* and *D. fragilis* detection, both methods showed high specificity but limited sensitivity, likely due to inadequate DNA extraction from the parasite. Molecular assays seem to be critical for the accurate diagnosis of *E. histolytica*. Overall, PCR results from preserved stool samples were better than those from fresh samples, likely due to better DNA preservation in the former.

**Conclusions:**

Molecular methods show promise for the diagnosis of intestinal protozoan infections. The molecular assays tested in this investigation performed well for *G. duodenalis* and *Cryptosporidium* spp. in fixed faecal specimens, while *D. fragilis* detection was inconsistent. These results suggest that although PCR techniques are promising in terms of reliable and cost-effective parasite identification, further standardisation of sample collection, storage and DNA extraction procedures is necessary for consistent results.

**Graphical Abstract:**

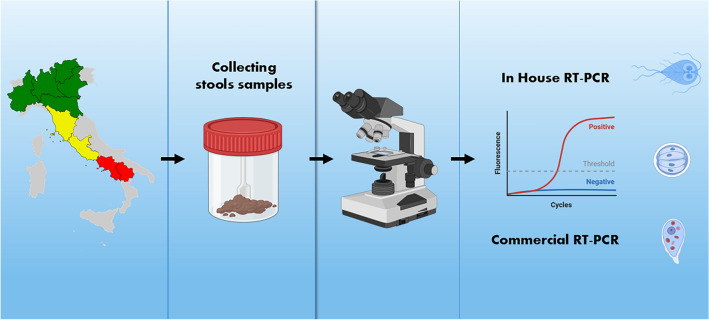

**Supplementary Information:**

The online version contains supplementary material available at 10.1186/s13071-025-06879-9.

## Background

Pathogenic intestinal protozoa exhibit a worldwide distribution and are among the leading etiological agents of diarrheal diseases [1]. It is estimated that intestinal protozoan parasites infect almost 3.5 billion people, causing approximately 1.7 billion episodes of diarrheal disorders annually [2]. Such infections typically arise from the consumption of food or drinking water contaminated by cysts or oocysts of these protozoa; alternative routes of infection may include contact with animals, the utilisation of recreational water sources, such as swimming pools [3, 4], and, more rarely, by sexually routes [5]. Diagnosing these infections poses a formidable challenge, even for experienced microbiologists [6]. The most frequently reported intestinal protozoan is *Giardia duodenalis* [7], with reports that is responsible for about 280 million symptomatic infections and 2.5 million deaths annually [8], followed by *Entamoeba histolytica* and *Cryptosporidium* spp. Taken together, these three pathogens represent the predominant protozoa implicated in diarrhea, contributing to a significant disease burden [9, 10]. The diagnosis of other intestinal protozoa, such as *Blastocystis hominis* and *Dientamoeba fragilis*, is largely neglected, thereby limiting our knowledge on their pathogenic relevance and impact on global health [11]. Nevertheless, numerous reports correlate these protozoa with human illness [12–14].

*Giardia duodenalis* infections cause diarrhea characterised by loose, greasy stools and flatulence. Weight loss is also frequently associated with *G. duodenalis* infection, whereas fever and other systemic symptoms are infrequent [15]. Among the *Entamoeba* species, infections in humans primarily are caused by non-pathogenic species, such as *E. dispar* and *E. coli*, and by a pathogenic species, *E. histolytica*. Although asymptomatic infections are the most common, 10% of infected subjects exhibit symptoms of invasive amoebiasis. In such cases, *E. histolytica* is responsible for dysentery due to ulceration of the colonic mucosa, which extends to the submucosa and results in bloody diarrhea. Moreover, *E. histolytica* has the potential to induce the formation of liver abscesses [16, 17]. The symptoms of *Cryptosporidium* spp. infection depend on the patient’s immune status. In immunocompetent individuals, the infection can be asymptomatic or manifest as self-limiting watery diarrhea. Conversely, immunocompromised patients, such as those living with human immunodeficiency virus (HIV) or those undergoing immunosuppressive therapy, may experience a spectrum of infections ranging from asymptomatic to fulminant infections. These patients may exhibit hepatobiliary complications and, more rarely, manifestations involving the respiratory tract [18, 19]. Patients infected with *D. fragilis* predominantly present with abdominal pain and diarrhea, but they may also experience weight loss, anorexia, flatulence, nausea, vomiting and anal pruritus; nonetheless, asymptomatic infections are common [20].

Microscopic examination of concentrated faecal specimens remains the reference method in clinical laboratories for the diagnosis of protozoan intestinal infections [21]. This method has the advantage of being a low-cost diagnostic method and as such useful in endemic areas characterised by high parasitic prevalence but low resources. However, microscopy-based diagnosis of protozoan infections requires qualified microscopists, is time-consuming and is characterised by significant limitations regarding sensitivity and specificity. Furthermore, the identification of related species using microscopic analysis alone may be misleading [22]. For example, it is impossible to differentiate cysts of non-pathogenic species of *Entamoeba* from the pathogenic *E. histolytica* by the microscopic examination [23]. Immunofluorescence microscopy shows greater sensitivity and specificity than traditional microscopy but is expensive and requires expert personnel [24].

In recent years, alternative diagnostic methodologies have emerged, such as immunochromatography and the enzyme-linked immunosorbent assay (ELISA), for the detection of intestinal protozoan infections. ELISA tests show considerable promise, primarily due to being simple to run and providing rapid screening. Nevertheless, ELISA tests frequently yield elevated rates of false positive and false negative results, thereby constraining their practical utility [25]. The issue of sensitivity is particularly critical in developed countries characterised by a low prevalence of infection. More recently, the employment of molecular diagnostic methods for the identification of intestinal protozoan infections has demonstrated increased sensitivity and specificity [26], prompting clinical laboratories to move from traditional approaches to molecular diagnostic techniques [27]. Over the years, several molecular methods have been developed based on real-time PCR (RT-PCR) assays, resulting in the improved sensitivity of diagnostic procedures and diminishing the necessity for multiple stool samples to be analysed [11]. Nevertheless, in contrast to tests developed for bacterial and viral pathogens, molecular methods aimed at detecting intestinal protozoan infections are still experiencing technical limitations since the robust wall structure of these organisms complicates the DNA extraction process from parasite oocysts. Therefore, while PCR assays offer a time-efficient solution for laboratory personnel and reduce the financial burden associated with diagnosing intestinal protozoa, some authors recommend molecular techniques as a complementary method rather than as a replacement of conventional microscopic methodologies, primarily because microscopic examination can reveal additional parasitic intestinal infections that are not targeted by PCR assays [28].

The multicentre study reported here was primarily designed to evaluate the performance of a commercial RT-PCR test (AusDiagnostics Company - R-Biopharm Group, Mascot, Australia) in conjunction with an in-house RT-PCR assay, which has been previously validated in the Microbiology Unit of Padua Hospital (Padua, Italy). We compared the performance of these molecular platforms for diagnosing intestinal protozoa from stool samples to the performance of the conventional microscopic reference method. This investigation aims to contribute to broader the body of existing literature on the utility of molecular assays in parasitology, particularly within non-endemic contexts.

## Methods

### Study design

**Fig. 1:**
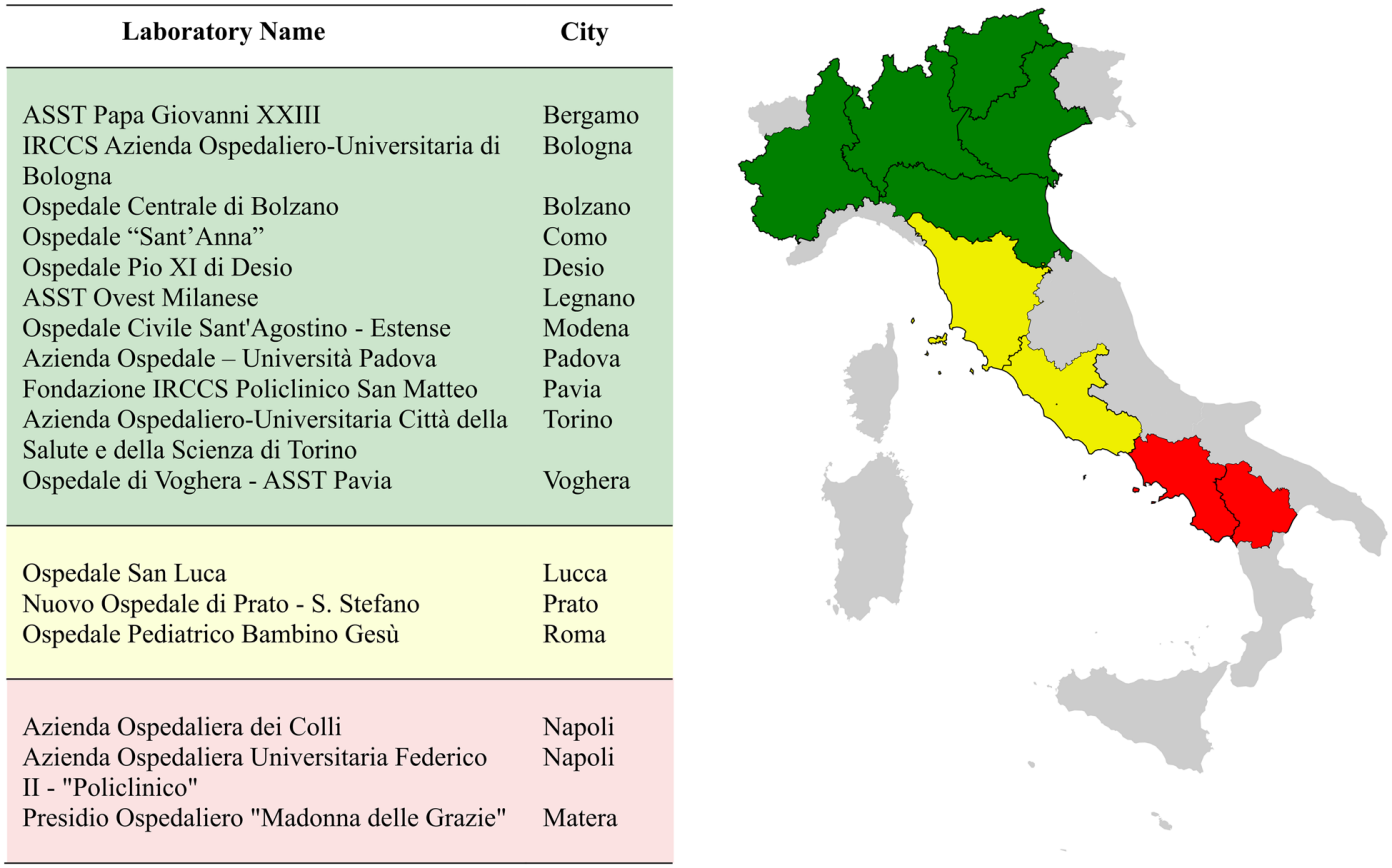
Geographic distribution of the laboratories participating in the study

**Fig. 2:**
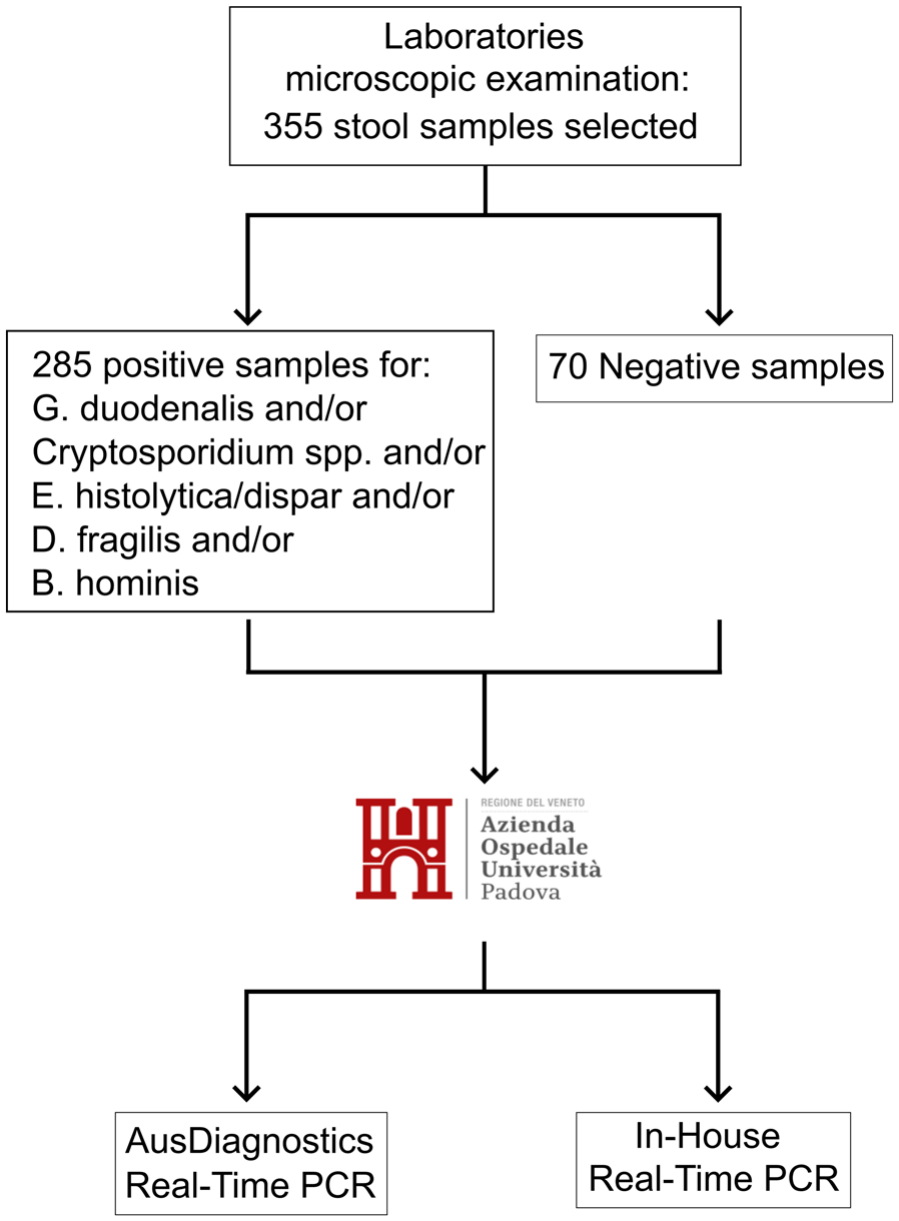
Schematic representation of the study flow chart

A total of 18 microbiological laboratories throughout Italy participated in this multicentre study. All participating laboratories voluntarily responded to an initiative put forth by the Committee for the Study of Parasitology of the Italian Association of Clinical Microbiologists (CoSP-AMCLI). Twelve laboratories were located in the northern regions of the country, three in the central area and three in the southern part (see Fig. 1). A total of 355 consecutive stool samples were collected over a span of 6 months by the participating laboratories. Of these, 230 stool samples were deemed fresh, whereas 125 were preserved in Para-Pak media (Fig. 2).

All 355 samples were examined using conventional microscopy in accordance with the guidelines issued by the WHO and U.S. Centres of Disease Control and Prevention (CDC) [29, 30]. Fresh stool samples were stained with Giemsa, while fixed samples were all processed using the FEA (formalin-ethyl acetate) concentration technique. Subsequent to the examination, all samples were promptly frozen and stored at − 20 °C.

The samples were sent to the UOC of Microbiology and Virology of Padua University Hospital for molecular study employing the commercial AusDiagnostics Company kit (distributed by Nuclear Laser Medicine, Milan, Italy) and an in-house molecular assay to detect *Giardia lamblia*, *Cryptosporidium *spp., *E. histolytica* and *D. fragilis*. Among the samples tested, 70 yielded negative results, whereas 285 tested positive using conventional microscopy for *G. lamblia*, *Cryptosporidium* spp., *E. histolytica*, *D. fragilis*, *Blastocystis hominis* and other commensal protozoa including *E. coli*, *Entamoeba hartmanni*, *Endolimax nana* and *Chilomastix mesnili*.

### DNA extraction

A volume of 350 µl of S.T.A.R (Stool Transport and Recovery Buffer; Roche Applied Sciences, Basel, Switzerland) was mixed with approximately 1 µl of each faecal sample using a sterile loop and incubated for 5 min at room temperature, following which the samples were centrifuged at 2000 rpm for 2 min. The supernatant (250 µl) was carefully collected, transferred to a fresh tube and combined with 50 µl of the internal extraction control. DNA was then extracted using the MagNA Pure 96 DNA and Viral NA Small Volume Kit on the MagNA Pure 96 System (Roche Applied Sciences), which is a fully automated nucleic acid preparation based on magnetic separation of nucleic acid-bead complexes.

### In-house RT-PCR amplification

Each reaction mixture included 5 µl of MagNA extraction suspension, 2× TaqMan® Fast Universal PCR Master Mix (12.5 µl) (Thermo Fisher Scientific, Waltham, MA, USA), primers and probe mix (2.5 µl) and sterile water to a final volume of 25 µl. A multiplex tandem PCR assay was performed using the ABI 7900HT Fast Real-Time PCR System (Applied Biosystems™, Thermo Fisher Scientific, USA), applying the following cycling regimen: 1 cycle of 95 °C for 10 min; followed by 45 cycles each of 95 °C for 15 s and 60 °C for 1 min. Each run included a positive control and at least one water blank as a negative control to exclude contamination. The luciferase gene served as an internal extraction control to validate the integrity of the DNA template for each sample. The primers and probes were designed to target specific DNA sequences from *G. duodenalis, Cryptosporidium* spp.*, E. histolytica, E. dispar* and *D. fragilis* and are listed in Table 1. This methodology was rigorously validated utilising positive controls provided by Quality Control for Molecular Diagnostics (QCMD; Glasgow, UK).

**Table 1:** Oligonucleotides and PCR conditions

Target organism	Oligonucleotide name	Final concentration	Oligonucleotide sequence	Size of the target region (bp)	Target gene
*Entamoeba histolytica* [40]	Ehd-239F	25 pM	5′-CATTAAAAATGGTGAGGTTCTTAGGAA-3′	172	SSU rRNA
	Ehd-88R	25 pM	5′-TGGTCGTCGTCTAGGCAAAATATT-3′		
	histolytica-96 T	5 pM	FAM 5′-TTGACCAATTTACACCGTTGATTTTCGGA-3′ EDQ		
*Entamoeba dispar* [40]	Ehd-239F	25 pM	5′-GGATCCTCCAAAAAATAAAGTTTTATCA-3′	172	SSU rRNA
	Ehd-88R	25 pM	5′-ATCCACAGAACGATATTGGATACCTAGTA-3′		
	dispar-96 T	5 pM	YY 5′-UGGUGAGGUUGUAGCAGAGAUAUUAAUU-3′ EDQ		
*Giardia lamblia* [41]	Giardia-80F	6.25 pM	5′-GACGGCTCAGGACAACGGTT-3′	62	SSU rRNA
	Giardia-127R	6.25 pM	5′-TTGCCAGCGGTGTCCG-3′		
	Giardia-105 T	2.5 pM	FAM 5′-CCCGCGGCGGTCCCTGCTAG-3′ BHQ1		
*Cryptosporidium * spp. [41]	CrF	25 pM	5′-CGCTTCTCTAGCCTTTCATGA-3′	138	DNAj-like protein
	CrR	25 pM	5′-CTTCACGTGTGTTTGCCAAT-3′		
	Crypto	8.75 pM	TR 5′-CCAATCACAGAATCATCAGAATCGACTGGTATC-3′ BHQ2		
*Dientamoeba fragilis* [42]	5DMB	15 μM	5′-GGCGAAAGCATCTATCAAGTGTATT-3′	101	16S-like rRNA
	3DMB	15 μM	5′-CGGCATCGTTTAAGGTAGGAAC-3′		
	DMBP	30 μM	FAM 5′-ACCCGGGTCTCTGATCCGGTTGG-3′ TAMRA		

### AusDiagnostics Company RT-PCR assay

HighPlex Systems are based on the multiplexed tandem PCR (MT-PCR) principle. The AusDiagnostics Company kit is designed to identify the DNA genome of *G. duodenalis* (18S ribosomal RNA [rRNA])*, Cryptosporidium* spp. (*Cryptosporidium* oocyst wall protein [COWP])*, E. histolytica* (18S RNA) and *D. fragilis* (18S RNA). Positive and negative controls were included in each run. The sensitivity and specificity of the AusDiagnostics Company kit, as reported by the manufacturer (https://www.ausdiagnostics.com) are summarised in Table 1 (Additional file 1: Text 1).

### Ethics statement

The study was approved by the Ethics Committee for Clinical Trials of the Province of Padua (protocol no. 0000555) on 7 January 2020. All procedures were performed in accordance with the relevant national guidelines and regulations.

### Statistics analysis

The proportion of the true positive results in the case group (sensitivity) and the proportion of the true negative results in the control group (specificity) was calculated for all diagnostic methods employed in the study (AusDiagnostics Company RT-PCR and in-house RT-PCR assay) for all parasites. The Cohen’s kappa (*κ*) test was applied to determine test agreement, with *κ* < 0 indicating no agreement; 0–0.20, slight agreement; 0.21–0.40, fair agreement; 0.41–0.60, moderate agreement; 0.61–0.80, substantial agreement; and 0.81–1, an almost perfect agreement.

## Results

The laboratories selected a total of 355 samples that were tested using conventional microscopic techniques (Fig. 2). Overall, microscopic examination revealed the presence of a single *G. duodenalis* infection in 78 stool samples, *Cryptosporidium* spp. in 18 stool samples, *D. fragilis* infection in 67 stool samples, *E. histolytica/dispar* infection in 21 stool samples and *Blastocystis hominis* infections in 62 stool samples. Additionally, 70 samples tested negative and 39 exhibited co-infections involving ≥ 2 parasites within a single host, as detailed in Table 2.

**Table 2:** Samples with multiple parasites detected by microscopic examination

Co-infection: *n* = 39				
Parasites identified: *n* = 84				
Number of samples	First parasite	Second parasite	Third parasite	Fourth parasite
22	*D. fragilis*	*Blastocystis hominis*		
5	*E. histolytica/dispar*	*B. hominis*		
2	*G. duodenalis*	*B. hominis*		
2	*G. duodenalis*	*B. hominis*	*D. fragilis*	
2	*G. duodenalis*	*D. fragilis*		
2	*G. duodenalis*	*E. histolytica/dispar*		
1	*G. duodenalis*	*Cryptosporidium* spp.		
1	*G. duodenalis*	*E. histolytica/dispar*	*D. fragilis*	*B. hominis*
1	*G. duodenalis*	*E. histolytica/dispar*	*B. hominis*	
1	*E. histolytica/dispar*	*D. fragilis*	*B. hominis*	

Due to the limited volume of each sample, it was not feasible to re-examine the samples when discrepancies appeared between the molecular and microscopic evaluations.

### *Giardia duodenalis* detection

Overall, 89 of the 355 stool samples tested positive for *G. duodenalis* by conventional microscopic examination (Table 3). The commercial molecular kit (AusDiagnostics Company) detected *G. duodenalis* in 81/355 stool samples (sensitivity: 91.0%; specificity: 98.9%), and the in-house RT-PCR detected *G. duodenalis* in 83/355 stool samples (sensitivity: 93.3%; specificity: 97%). The commercial kit yielded eight false negatives and three false positives, whereas the in-house assay yielded six false negatives and eight false positives.

**Table 3:** Overall performance of the AusDiagnostics kit and the in-house real-time PCR kit to detect *G. duodenalis*, *Cryptosporidium* spp., *D. fragilis* and *E. histolytica/dispar*

Parasite	Sensitivity (%)		Specificity (%)	
	AusDiagnostics kit	In-house RT-PCR	AusDiagnostics kit	In-house RT-PCR
*G. duodenalis*	81/89 (91.0%)	83/89 (93.3%)	263/266 (98.9%)	258/266 (97.0%)
*Cryptosporidium* spp*.*	15/19 (78.9%)	15/19 (78.9%)	333/336 (99.1%)	336/336 (100%)
*D. fragilis*	65/95 (68.4%)	65/95 (68.4%)	239/260 (91.9%)	240/270 (88.9%)
*E. histolytica/dispar*	nd^a^	nd^a^	nd^a^	nd^a^

The numerator in the fraction is the number of positive stool samples detected using the AusDiagnostics Company kit or the in-house RT-PCR kit, respectively; the denominator is the number of positive stool samples detected by conventional microscopic examination. The sensitivity and specificity are given in parentheses following the fraction

RT-PCR Real-time PCR

^a^nd: Sensitivity and specificity not determined for *E. histolytica* because microscopy does not enable identification to the species level

We next determined the impact of the characteristics of the stool samples on the performance of the molecular methods. Of the 230 fresh stool samples tested, 66 were positive for *G. duodenalis* by conventional microscopy (Table 4). Among these, 61/230 samples (sensitivity: 89.4%; specificity: 98.8%; *κ*: 0.90) and 65/230 samples (sensitivity: 90.9%; specificity: 96.9; *κ*: 0.88) tested positive using the commercial and in-house molecular assays, respectively (Table 4). In the case of fresh stools, the commercial kit yielded seven false negatives and two false positives, while there were six false negatives and five false positives using the in-house assay.

**Table 4:** Performance of the AusDiagnostics Company kit and the in-house real-time PCR kit to detect *G. duodenalis*, *Cryptosporidium* spp., *D. fragilis* and *E. histolytica/dispar* in fresh stool samples

Fresh stool samples:* n* = 230				
Parasite	Sensitivity (%)		Specificity (%)	
	AusDiagnostics kit	In-house RT-PCR	AusDiagnostics kit	In-house RT-PCR
*G. duodenalis*	59/66 (89.4%)	60/66 (90.9%)	162/164 (98.8%)	159/164 (96.9%)
*Cryptosporidium* spp*.*	12/16 (75%)	12/16 (75%)	211/214 (98.6%)	214/214 (100%)
*D. fragilis*	41/64 (64.0%)	41/64 (64.0%)	152/166 (91.6%)	153/166 (92.2%)
*E. histolytica/dispar*	nd^a^	nd^a^	nd^a^	nd^a^

The numerator in the fraction is the number of positive stool samples detected using the AusDiagnostics Company kit or the in-house RT-PCR kit, respectively; the denominator is the number of positive stool samples detected by conventional microscopic examination. The sensitivity and specificity are given in parentheses following the fraction

RT-PCR Real-time PCR

^a^nd: Sensitivity and specificity not determined for *E. histolytica* because microscopy does not enable identification to the species level

Conversely, 23/125 preserved stool samples tested positive for *G. duodenalis* via conventional microscopy (Table 5). Among preserved specimens, 23/125 samples were confirmed to be *G. duodenalis*-positive using the commercial assay (sensitivity: 95.6%; specificity: 99%; *k*: 0.94), and 26/125 samples were confirmed to be *G. duodenalis*-positive using the in-house molecular assay (sensitivity: 100%; specificity: 97.1%; *κ*: 0.92). For preserved stools, the commercial kit yielded one false negative and one false positive, whereas there were three false positives using the in-house assay.

**Table 5:** Performance of the AusDiagnostics Company kit and the in-house real-time PCR kit to detect *G. duodenalis*, *Cryptosporidium* spp., *D. fragilis* and *E. histolytica/dispar* in preserved stool samples

Preserved stool samples:* n* = 125				
Parasite	Sensitivity (%)		Specificity (%)	
	AusDiagnostics kit	In-house RT-PCR	AusDiagnostics kit	In-house RT-PCR
*G. duodenalis*	22/23 (95.6%)	23/23 (100%)	101/102 (99.0%)	99/102 (97.1%)
*Cryptosporidium* spp*.*	3/3 (100%)	3/3 (100%)	122/122 (100%)	122/122 (100%)
*D. fragilis*	24/31 (77.4%)	24/31 (77.4%)	87/94 (92.6%)	87/94 (92.6%)
*E. histolytica/dispar*	nd^a^	nd^a^	nd^a^	nd^a^

The numerator in the fraction is the number of positive stool samples detected using the AusDiagnostics Company kit or the in-house RT-PCR kit, respectively; the denominator is the number of positive stool samples detected by conventional microscopic examination. The sensitivity and specificity are given in parentheses following the fraction

RT-PCR Real-time PCR

^a^nd: sensitivity and specificity not determined for *E. histolytica* because microscopy does not enable identification to the species level

The comparison between the two molecular tests showed an excellent level of agreement (*κ* > 0.8).

### *Cryptosporidium* spp. detection

Of the 355 samples tested for *Cryptosporidium* spp., 19 tested positive by conventional microscopic examination, of which 15/355 were confirmed as positive by both molecular methods (AusDiagnostics Company kit and in-house RT-PCR), demonstrating an identical sensitivity of 78.9% compared to the reference method (Table 3). The specificity for the detection of *Cryptosporidium* spp. was recorded at 99.1% for AusDiagnostics Company kit and 100% for the in-house assay.

For freshly preserved stool samples, 16/230 were identified as positive for *Cryptosporidium* spp. by conventional microscopy (Table 4). Among these, 15/230 samples (sensitivity: 75%; specificity: 98.6%; *κ*: 0.76) and 12/230 samples (sensitivity: 75%; specificity: 100%; *κ*: 0.85) tested positive using the commercial and in-house molecular assays, respectively (Table 4). The commercial RT-PCR kit for fresh stool samples yielded three false positives and four false negatives, while the in-house assay resulted in four false negatives without any false positives.

Conversely, 3/125 preserved stool samples yielded positive results for *Cryptosporidium* spp. by conventional microscopy (Table 5). Among the preserved specimens, all three *Cryptosporidium* spp. detected by microscopic examination in preserved stool samples were successfully identified by both molecular techniques (sensitivity: 100%; specificity: 100%; *κ*: 1).

Notably, of the 16 *Cryptosporidium* spp. detected in the fresh stool samples by traditional microscopic examination, only 12 were recognised by the two molecular methods, whereas all of the parasites identified in preserved stools were detected by the molecular methods.

The comparison between the two molecular tests showed an excellent level of agreement (*κ* > 0.8).

### *Dientamoeba fragilis* detection

Of the 355 stool samples tested, 95 samples tested positive for *D. fragilis* by conventional microscopic examination, of which 65 were confirmed as positive by both molecular methods (AusDiagnostics Company kit and in-house RT-PCR), demonstrating an identical sensitivity of 68.4% compared to the reference method (Table 3). The specificity for the detection of *D. fragilis* was recorded at 91.9% for AusDiagnostics Company kit and 88.9% for the in-house assay. The commercial molecular kit yielded 30 false negatives and 21 false positives, whereas the In-House RT-PCR yielded 20 false negatives and 30 false positives.

Of the fresh stool specimens, 64/230 were identified as positive for *D. fragilis* by conventional microscopy (Table 4). Among these, 55/230 samples (sensitivity: 64.0%; specificity: 91.6%; *κ*: 0.58) and 54/230 samples (sensitivity: 64.0%; specificity: 92.2%; *κ*: 0.59) tested positive using the commercial and in-house molecular assays, respectively (Table 4). The commercial molecular kit generated 14 false positives and 23 false negatives, whereas the In-House assay yielded 13 false positives and 23 false negatives for fresh stool specimens.

In comparison, of the preserved stool samples, 31/125 exhibited positive results for *D. fragilis* by conventional microscopy (Table 5). Among the preserved specimens, 31/125 *D. fragilis* were successfully identified through both molecular techniques (sensitivity: 77.4%; specificity: 92.6%; *κ*: 0.7). For preserved stool samples, the commercial RT-PCR kit and the in-house assay each yielded seven false positives and seven false negatives.

The comparison between the two molecular tests showed an excellent level of agreement (*κ* > 0.8).

### *Entamoeba histolytica/dispar* detection

Conventional microscopic examination fails to differentiate between the species *E. histolytica* and *E. dispar*; consequently, neither sensitivity nor specificity of onventional microscopic examination can be ascertained.

Overall, microscopic evaluation identified 31 (8.7%) stool samples as positive and 324 as negative for *E. histolytica/dispar*. Among the 31 samples deemed to be positive by microscopy, the in-house PCR assay detected *E. histolytica* in four samples and *E. dispar* in 12 samples. In contrast, the commercial kit identified only two samples as positive for *E. histolytica*, since it lacks a target for *E. dispar*.

Among the 324 samples that were negative by microscopy, the in-house assay detected two samples as positive for *E. dispar*. Both molecular methodologies concurred with the microscopy results, confirming the absence of additional *E. histolytica*.

## Discussion

Parasites contribute significantly to the global burden of disease [31]. The current gold standard for diagnosing intestinal parasitic infections is the traditional microscopic examination of stool samples, which aims to detect oocysts, cysts, trophozoites and ova of helminths. This methodology requires a time-consuming preparation process and is heavily reliant upon the observer's expertise, thereby demanding a high level of technical proficiency [22]. In light of the constraints associated with conventional microscopic techniques and the complexities inherent in molecular procedures, parasitologists advocate for the adoption of genomic amplification methods as a complementary diagnostic approach. Achieving consensus regarding the characteristics of molecular methods is pivotal for their integration into routine diagnostic workflows [32]. In this study, we evaluated the efficacy of two PCR assays for the detection of intestinal protozoa in comparison to that of conventional microscopic examination.

Our data reveal a remarkable concordance between the AusDiagnostics Company kit and in-house PCR methodologies for the detection of *G. duodenalis,* particularly in terms of the sensitivity and specificity*.* In our study, both molecular techniques demonstrated optimal alignment, with elevated sensitivity and specificity*,* comparable to those of conventional microscopic examination. These findings align with the results obtained in published studies, which report an average sensitivity and specificity of 95% and 93%, respectively, for the detection of *G. duodenalis* utilising molecular assays [33].

For *Cryptosporidium* spp., both molecular methodologies yield similar results, showing optimal agreement, with high specificity but limited sensitivity. These findings are partially congruent with those from previous investigations that found variable sensitivity and specificity in the detection of *Cryptosporidium parvum/C. hominis* by molecular assays [34]. The diminished sensitivity of molecular methods in comparison to microscopic examination may be attributable to inadequate DNA extraction, potentially due to the rigidity of the parasite or a low parasite burden within the faecal specimen [35]. Consequently, sample homogenisation and faecal preprocessing emerge as critical steps for obtaining sufficient parasite DNA.

The molecular methodologies used to detect *D. fragilis* exhibited moderate to substantial agreement, albeit with comparably inadequate sensitivity but high specificity, both consistent with recent reports [12]. While these observations may suggest insufficient DNA extraction from stool specimens, they warrant careful scrutiny, given that the reference standard of microscopic examination on wet preparations is inherently imprecise for this parasite. Traditional microscopic assessment encounters significant challenges to identifying *D. fragilis* trophozoites due to their rapid deterioration outside the intestinal lumen and the fragility of their binucleate structure [36]. Therefore, despite its limitations, PCR should be regarded as the definitive reference method for diagnosing dientamoebiasis [37]. Regarding the detection of *E. histolytica*, the two molecular assays tested in the present study yielded consistent results, as the positive stool specimens came from patients diagnosed with the *Entamoeba complex* based on microscopy. Given that microscopy fails to differentiate between pathogenic and non-pathogenic* Entamoeba* species, the sensitivity and specificity of the molecular assays were not determined. Therefore, for future applications, molecular biology methods must be able to discern between *E. histolytica* and the carriage of the non-pathogenic* Entamoeba* complex (i.e. *E. dispar*, *E. moshkovski* or *E. bangladeshi*), thereby ensuring accurate identification and mitigating the risk of misdiagnosis [38].

Although our study was not specifically designed to assess the efficacy of the two molecular methodologies (AusDiagnostics Company kit and in-house RT-PCR) according to the various treatments of the stool samples, we observed that both molecular methodologies yielded superior results with fixed faecal samples in comparison to their fresh counterparts for all tested parasites. It is conceivable that preserving the integrity of parasites in fixed stools enhances the availability of high-quality DNA for PCR analysis. Moreover, the inhibition of DNAse activity in fixed faecal specimens mitigates DNA degradation, thereby augmenting the success rate of molecular assays [40].

Although molecular techniques for detecting faecal parasites exhibit remarkable potential to surpass conventional microscopic methodologies, each phase preceding amplification still reveals specific weaknesses [38]. The most critical step influencing the outcome of molecular-based detection of intestinal parasites is the DNA extraction process, which is crucial for yielding sufficient quantities of high-quality, pure DNA. Faecal components, such as bile salts, bilirubins and carbohydrates, may impede polymerase activity [40]. Additionally, the genetic material of protozoa, mainly located within oocysts that have robust cell walls, can hinder DNA isolation [41]. Consequently, both manual and automated extraction systems are equally affected by the mechanical disruption of faecal specimens [42]. This study was not designed to evaluate different DNA extraction protocols; therefore, all specimens were extracted utilising the MagNA technology without prior sample treatment. This methodology, particularly for *G. duodenalis* and *Cryptosporidium spp*., yielded suitable DNA for successful PCR, especially when preserved stool specimens were used. In light of these challenges, a primary objective within the field of clinical parasitology should be to establish validated DNA extraction protocols that are specifically tailored for diverse intestinal parasites.

Our study has several limitations. The foremost constraints pertain to the relatively modest sample size and the heterogeneous characteristics of the stool specimens analysed, which included both fresh and preserved samples. Furthermore, we were unable to access the patients’ clinical records, which precluded the inclusion of critical demographic information such as age, sex and comorbidities. Another potential confounding variable arises from the study's reliance on conventional microscopic examination as the reference standard for diagnosing parasitic infections, despite the well-documented variability in sensitivity that is dependent on the microscopist's expertise. Additionally, we could not ascertain whether the samples that tested positive by molecular assays but negative upon microscopic examination were genuinely positive, as we lacked the means to sequence the amplicons. Moreover, we did not employ a mechanical preprocessing protocol for the fresh and fixed samples to enhance the efficacy of DNA extraction.

## Conclusions

In conclusion, the accurate and fast detection of clinically significant enteric protozoa is highly desirable for a prompt, adequate and effective intervention [43]. The two molecular assays tested in the present study (AusDiagnostics Company kit and in-house RT-PCR) exhibited remarkable efficacy in utilising fixed faecal specimens for the detection of *G. duodenalis* and *Cryptosporidium spp*., although the identification of *D. fragilis* remains inconsistent. While PCR techniques are gaining increased attention in diagnostic laboratories for the development of reliable and cost-effective methods to identify faecal parasites, further studies are needed to standardise procedures for sample collection, storage and DNA extraction, as these pivotal steps are essential for achieving consistent results.

## Supplementary Information

Additional file 1: Text 1. Sensitivity and specificity of the AusDiagnostics Company kit, as reported by the manufacturer

## Data Availability

The data supporting the findings of the study must be available within the article and/or its supplementary materials, or deposited in a publicly available database.
