# Supplementary material for: Comparative analysis of commercial and “In-House” molecular tests for the detection of intestinal protozoa in stool samples
Source: Parasit Vectors. 2025 Aug 1;18:320. doi: 10.1186/s13071-025-06879-9 (PMC12317615; doi:10.1186/s13071-025-06879-9)
Supplement: Supplementary file 1 — Additional file 1: Text 1. Sensitivity and specificity of the AusDiagnostics Company kit, as reported by the manufacturer [file 13071_2025_6879_MOESM1_ESM.docx]

**Table 1** Performance claimed by AusDiagnostics Company kit

| **Assay** | Sensitivity % | Specificity % |
| --- | --- | --- |
| *Giardia duodenalis* (18S) | 98.6 % | 99.6 % |
| *Cryptosporidium spp.* | 97.8 % | 99.8 % |
| *D. fragilis* | 100 % | 100 % |
| *E. histolytica* | 91.7 % | 100 % |
